# Scenic area attractiveness in Dali City and its influencing factors evaluated using multi-source spatiotemporal data

**DOI:** 10.1371/journal.pone.0323310

**Published:** 2025-05-15

**Authors:** Di Wang, Haizhong Qian

**Affiliations:** Institute of Geospatial Information, Information Engineering University, Zhengzhou, China; Macau University of Science and Technology, MACAO

## Abstract

Scenic area attractiveness is a core factor in urban tourism development. Developments in social media and multi-source spatiotemporal data provide a basis for studying complex tourist behaviors, overcoming the limitations of traditional interview survey data. This study combines point of interest (POI), mobile signaling, and microblog check-in data to analyze scenic area popularity in Dali using kernel density analysis, hotspot analysis, and gravity models. It also uses ROST-CM6 to perform sentiment analysis on microblog check-in and text data to obtain tourist satisfaction, and combines the popularity and satisfaction to assess scenic area attractiveness. Additionally, GeoDetector is used to examine the impact of subjective human factors, objective factors of the attractions themselves, and the number of POI facilities around the attractions on the scenic area attractiveness in Dali. We obtained several key findings. First, the distribution of scenic areas in Dali City showed a two-center, multi-point pattern, including two core scenic areas (i.e., Dali Ancient City and Xizhou Ancient Town) and numerous scattered areas. Second, the majority of scenic areas in Dali City were more active in the daytime than at night, whereas Dali Ancient City was most active at night. Tourists in Dali City mostly came from Yunnan Province, neighboring provinces, and economically developed coastal regions. Third, a text-based sentiment analysis revealed numerous high-frequency adjectives reflecting positive sentiment, indicating high scenic area satisfaction. Fourth, the number of internal POIs had the greatest effects on scenic area popularity and attractiveness. Specifically, the more POIs, the more popular and attractive the scenic area. The interactive decision-making power of various factors was greater than the decision-making power of individual factors. These findings provide insight into the determinants of scenic area satisfaction, providing a basis for the development of urban tourism.

## Introduction

Scenic area attractiveness refers to the degree to which tourists are excited by the diverse and varied tourism resources in a scenic area. It is of crucial importance to tourist destinations and can partially reflect the level of tourism development. Scenic area attractiveness can be evaluated in terms of the characteristics, value, function, combination, structure, and scale of tourism resources. Located in the southwest border of China, Dali City is a tourist city known for its natural scenery and unique cultural and historical resources. In 2016, Dali City was listed in the first batch of “National All-for-one Tourism Demonstration Zones.” Continuous tourism development in Dali City has faced challenges with respect to the upgrade of urban infrastructure, innovation of the tourism product supply, management of tourism market environment, and protection of the ecological environment. Therefore, it is important to examine the scenic area attractiveness of Dali City and its influencing factors.

Initial studies of scenic area attractiveness abroad have mainly focused on concept definition, modeling, and influencing factors. With respect to concept definition, has been described as a type of push force [1], a collection of destination attributes and characteristics [2], and a combination of natural attractiveness and perceptions of tourists [3]. With respect to modeling, Crampon was the first to construct a tourism attractiveness model based on the visit count, tourism source-destination distance, and economic development of source regions [4], and this model was subsequently modified by Wolfe [5]. Chang et al. constructed a model for tourism attractiveness based on the fuzzy set theory [6]. Barros et al. presented a tourism attractiveness customer satisfaction index (TACSI) [7]. Harandi et al. constructed a five-dimension gravity model for health tourism using the inductive coding approach [8]. Additionally, various factors influence scenic area attractiveness, including tourist cognition and spatial distance [9], a combination of spatial distance, tourism resources and products, and innovation [10], personalized services [11], and natural conditions and the hotel environment [12]. Boivin et al. found that the majority of tourists perceive tourism attractiveness based on the urban environment and public space [13].

Local studies of scenic area attractiveness have predominantly focused on gravity model construction, influencing factors, and evaluation of tourism attractiveness. Zhang constructed a tourism gravity model based on the mechanism underlying tourism attractiveness [14]; this model was extended by Bao and Ma et al. [15]. Wan modified the Grampon model [16], Wang et al. created a tourism gravity model based on fuzzy mathematics [17], and Li constructed a gravity model based on the Wilson paradigm [18]. Tian et al. developed an attractiveness evaluation index system, covering various factors, such as tourism resources, scenic area infrastructure, regional characteristics, and image promotion [19]. Shi et al. analyzed the effects of theme, product experience, locational conditions, admission tickets, and infrastructure improvement [20]. Zhang analyzed the influence of historical buildings, historical and cultural landscape, religious and cultural landscape, commercial street culture, and recreational culture [21]. Xu et al. discussed the effects of diverse factors (e.g., natural conditions, urban infrastructure, and socioeconomic development) on the distribution of regional tourism resources [22]. Wang et al. examined the factors influencing the spatial distribution of scenic areas, including geographic landscape, cultural heritage, socioeconomic development, and transportation conditions [23]. Attractiveness evaluation systems have been developed for revolutionary scenic areas [24–26], urban parks [27], ethnic cultural tourism [28–30], and the rural tourism landscape [31,32].

With rapid technological advancements, large-scale mobile positioning data with high spatiotemporal accuracy and social media check-in data provide new sources of data for studying human activities. There are three main types of big data in tourism research: electricized traditional data, multi-source heterogeneous user-generated content, and spatiotemporal behavioral data generated via tourism activities [33]. Relevant studies have covered the following: 1) spatial distribution patterns based on POI data [34–36]; 2) sentiment analyses of POIs based on online comment data [37,38]; 3) characteristics of urban scenic areas based on microblogging data [39–42]; 4) spatiotemporal distribution of tourists based on mobile signaling data [43–45]; and 5) tourist evaluation models for POIs and destinations based on user-generated content [46–49].

Multi-source spatiotemporal data provides a new analytical paradigm for tourism research, yet existing studies still have limitations in data fusion and spatiotemporal dynamic analysis. Although social media data and POI data have been widely used in tourist behavior studies, current methods often rely on static analysis of data sources, making it difficult to capture the spatiotemporal dynamic characteristics of tourist behavior, such as diurnal flow fluctuations and the migration of hotspot areas. Mobile signaling data, as a high-precision and continuous spatiotemporal behavior recording medium, can accurately depict tourists’ spatial locations, dwell times, and movement patterns. However, existing research has yet to fully explore its potential in the analysis of scenic area attractiveness. Therefore, based on existing literature, this study combines POI, mobile signaling, and microblog check-in data, approaching the analysis of scenic area attractiveness from two dimensions: popularity and satisfaction. By adopting multi-source data fusion and spatiotemporal behavior analysis methods, this study provides a comprehensive analysis of the scenic area attractiveness in Dali. Additionally, it further examines the multiple influencing factors of scenic area attractiveness. Through this innovative method, this study can accurately capture the temporal and spatial changes of tourist behavior, and provides a new perspective for the application of multi-source data fusion in the research of scenic attraction, and provides data support and decision-making basis as well as a new framework for quantitative evaluation for resource optimization, tourist management, regional coordination and sustainable growth of Dali’s tourism industry, so as to promote Dali to maintain its advantages in the increasingly competitive tourism market. And ensure the long-term health of tourism.

## Materials and methods

### Overview of Dali City

**Fig 1:**
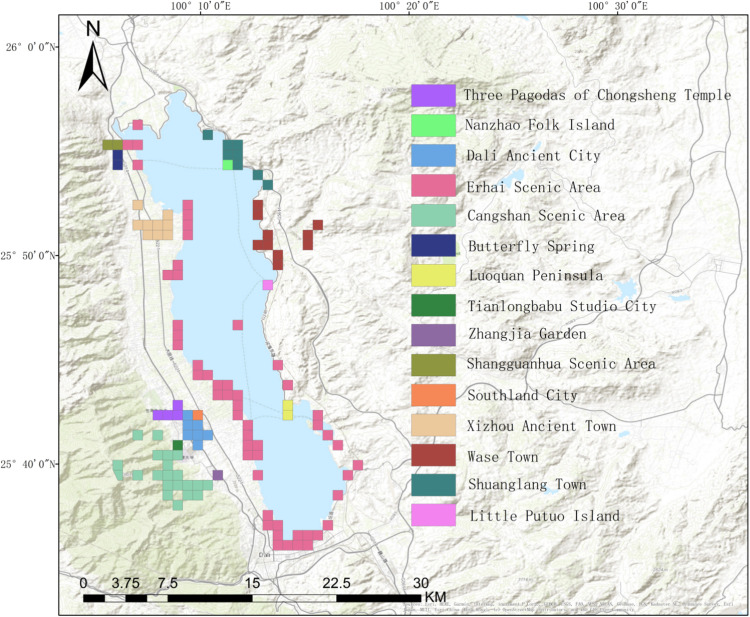
Distribution map of scenic spots.

Dali City, which has a long development history in southwestern China, is an important tourist destination in Yunnan Province. Dali City boasts densely distributed tourism resources and well-developed tourism facilities, including historical and cultural heritages represented by Dali Ancient City, natural landscape represented by Cangshan Mountain and Erhai Lake, Bai ethnic villages represented by Xizhou Ancient Town and Shuanglang Town, and urban cultural resources represented by Xiaguan Town. In this study, Dali City was selected to examine urban scenic area attractiveness owing to its diverse types of scenic areas, large number of tourists, high degree of informatization, and high Internet penetration rate. To facilitate the acquisition of mobile signaling data, the study region was divided into numerous grids and scenic areas were represented as grids according to their actual size. Fig 1 shows the location of the study region and distribution of scenic areas.

### Data and methodology

**Fig 2:**
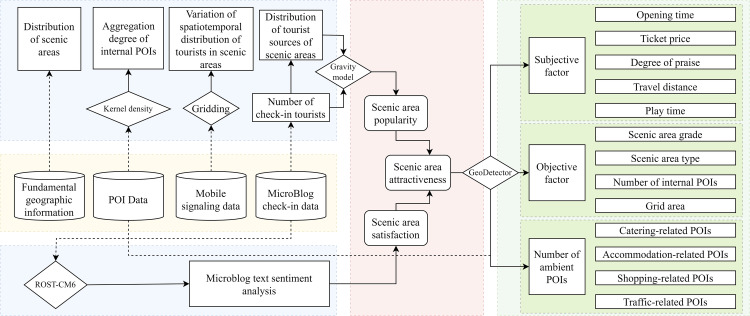
Technical roadmap of this study.

Fig 2 shows the complete study framework. First, we acquired and processed basic POI data, mobile signaling data for tourists, and microblog check-in data in the same period. Based on the data for scenic area aggregation and the spatiotemporal distribution of tourists, we then determined scenic area popularity using a gravity model, determined scenic area satisfaction based on microblog check-in text data, and determined scenic area attractiveness. Finally, we assessed the influencing factors using GeoDetector.

### Data sources and processing

To reduce the influence of crowd movement in non-scenic areas in Dali City on scenic area attractiveness, mobile signaling grid data were spatially connected with the POI data for scenic areas, and the data for effective POIs in the above 15 scenic areas and grids containing effective POIs were retained. The microblog check-in data with a “Blank” or “Miscellaneous” source were removed, and the data with check-in locations outside of scenic areas were also removed. Table 1 describes the details of the preprocessed data.

**Table 1:** Data sources and preprocessing.

Data	Source	Time	Data type	Experimental data
Mobile signaling data	China Unicom Wisdom Footprint Data Technology Co., Ltd.	June 12, 2023	Population in a 0.8 × 0.88 km grid within 24 h	123 grids after data preprocessing
Microblog check-in data	API of Sina microblog	January 1, 2023 to June 30, 2023	The check-in place contains the word “Dali”	14,444 original data records, 13,541 data records within the administrative region, and 4,822 effective data records after data preprocessing.
POI data	Gaode map crawler	2023	Scenic resort	671 original POIs and 397 effective POIs within scenic areas.

According to information on Dali City’s scenic areas released by www.China.com.cn combined with the spatiotemporal data of crowd movement, mobile signaling grid data, microblog check-in locations, and the POI distribution, tourism resources for Dali City were partitioned and classified into five grades (including 5A, 4A, 3A, 2A, and Distinctive), with a total of 15 scenic areas (Table 2). Among them, the A-level tourism area is authorized by the China National Scenic Area Quality Rating Committee to the provincial Tourism Administration, in accordance with the national standards of the “Management Measures for the quality of Tourist attractions” to evaluate the important signs of measuring the quality of scenic spots, from high to low is divided into 5 levels. 5A represents the highest level of national scenic spots, usually with very high tourist attraction and relatively perfect facilities and services; 4A is the second, still with high attraction and infrastructure; 3A is a medium grade, which meets basic tourism needs; 2A is a lower level scenic spot, mainly to meet the needs of specific tourists; Characteristic scenic spots refer to those that have certain local characteristics and uniqueness, but do not belong to any A-level scenic spots.

**Table 2:** Classification of scenic areas in Dali City.

Scenic Area	Grade	Scenic Area	Grade	Scenic Area	Grade
Three Pagodas of Chongsheng Temple	5A	Butterfly Spring	3A	Southland City	2A
Nanzhao Folk Island	4A	Luoquan Peninsula	3A	Xizhou Ancient Town	Distinctive
Dali Ancient City	4A	Tianlongbabu Studio City	3A	Wase Town	Distinctive
Erhai Scenic Area	4A	Zhangjia Garden	3A	Shuanglang Town	Distinctive
Cangshan Scenic Area	4A	Shangguanhua Scenic Area	3A	Little Putuo Island	Distinctive

### Methodology

#### Kernel density analysis.

Kernel density analyses are used to calculate the density of an element in its surrounding neighborhood. The kernel function is used to calculate the number of internal POIs per unit area, reflecting the relative spatial concentration of point elements. Each point is fitted into a smooth coniform surface to determine the main aggregation area and degree of POIs in each scenic area in a city.


$$f_{n}(x)=\frac{1}{nh}\sum_{i=1}^{n}k(\frac{x-x_{i}}{h})$$ (1)

where $f_{n}(x)$ denotes the kernel density function of location x of an element; n denotes the sample size; h denotes the search radius; k denotes the kernel function; $x_{i}$ denotes the location of an element within an area centered on location x.

#### Gravity model for scenic areas and tourist sources.

Given the long-term spatial interactions between scenic areas and tourist sources, Grampon [4] proposed the initial tourism gravity model:


$$T_{ij}=G(P_{i}A_{j}/D_{ij}^{b})$$ (2)

where $T_{ij}$ denotes the gravity between the tourist source i and destination j; G and b are empirical parameters; $P_{i}\;and\;A_{j}$ denote the emissivity of the tourist source and attracting capacity of the destination, respectively; $D_{ij}$ denotes the distance between the tourist source and destination. Improved models have been proposed, including Wilson’s modified model based on the principle of entropy maximization:


$$T_{ij}=P_{i}A_{j}exp(-\lambda c_{ij})$$ (3)

where $T_{ij}$ denotes the intensity of gravity between regions i and j; $P_{i}\;and\;A_{j}$ denote the economic intensity in regions i and j, respectively; λ denotes the damping coefficient; $c_{ij}$ denotes the generalized distance between regions i and j. By constructing a mechanistic model based on the principle of things, the distance decay model in the form of an exponential function offers an effective solution to the breakpoint paradox.

Based on empirical knowledge of tourism spatial interactions, scenic area attractiveness, “emissivity” of tourist sources, and “spatial damping” between tourist sources and scenic areas were included as basic explanatory variables in the tourism gravity model. A basic tourism gravity model was constructed using Equations (4) and (5):


$$T_{ij}=A_{j}P_{i}C_{i}exp(-\lambda r_{ij})$$ (4)

where, $T_{ij}$ denotes the spatial interaction between the tourist source i and scenic area j; $A_{j}$ denotes scenic area attractiveness; $P_{i}\;and\;C_{i}$ denote the emissivity of tourist sources; $P_{i}$ denotes the population size of region i; $C_{i}$ denotes the per-capita income level of region j; $r_{ij}$ denotes the generalized distance between tourist source i and scenic area j; λ denotes the spatial damping coefficient, which is positively correlated with the decay rate of spatial interaction (where the larger the λ value, the higher the decay rate). Using Equation (4), the following gravity model for scenic area attractiveness was generated:


$$A_{j}=\frac{\sum_{i}\frac{T_{ij}}{P_{i}C_{i}exp(-\lambda r_{ij})}}{i}$$ (5)

where $T_{j}$ denotes the total number of tourists in a scenic area. To determine the λ value, a relational equation was constructed [1] to calculate the damping coefficient under four scales (i.e., provincial, prefecture/city, county, and town scales). Considering the gravity between scenic areas and provincial-scale space, in the present study, the distance between Dali City and a tourist source was adopted as the generalized distance, and the damping coefficient was set to 0.00044 [18].

#### GeoDetector.

GeoDetector is a new statistical method for detecting spatial heterogeneity and ascertaining its driving factors [50]. Compared with classical statistical regression algorithms, GeoDetector has the ability to establish relationships between variables by discretizing the independent variable X; it is capable of detecting two-variable interaction effects while calculating the Q values of individual factors. This study included fewer than 30 datasets and therefore GeoDetector was suitable for analyses.

Factor_detector was used to detect the effect of a geographic factor on spatial heterogeneity, discretize the decision-making factor, and calculate its explanatory power Q for the index under different categories and in the whole study region as follows (Equation 6):


$$Q_{F,A}=1-\frac{1}{N\sigma_{A}^{2}}\sum_{i=1}^{m}N_{F,i}\sigma_{A_{F,i}}^{2}$$ (6)

where F denotes the decision-making factor; A denotes the attractiveness index; $Q_{F,A}$ denotes the explanatory power of F for A; $N\;and\;\sigma_{A}^{2}$ denote the sample size and variance in scenic area attractiveness, respectively; $N_{F,i}\;and\;\sigma_{A_{F,i}}^{2}$ denote the sample size and variance of the factor F under the category i, respectively; m denotes the number of categories for a decision-making factor. The value range of $Q_{F,A}$ is [0, 1]; the larger its value, the stronger its explanatory power for scenic area attractiveness.

Using interaction_detector, the interactive effects of different decision-making factors on scenic area attractiveness were evaluated. Five types of interactions were defined as follows:


$$Q(F_{1}\cap F_{2})<Min\left(Q\left(F_{1}\right),Q\left(F_{2}\right)\right)\;Nonlinear\;decay$$

$$Min\left(Q\left(F_{1}\right),Q\left(F_{2}\right)\right)<Q\left(F_{1}\cap F_{2}\right)<Max\left(Q\left(F_{1}\right),Q\left(F_{2}\right)\right)\;Single-factor\;nonlinear\;decay$$

$$Q\left(F_{1}\cap F_{2}\right)>Max\left(Q\left(F_{1}\right),Q\left(F_{2}\right)\right)\;Two-factor\;enhancement$$

$$Q\left(F_{1}\cap F_{2}\right)=Q\left(F_{1}\right)+Q\left(F_{2}\right)\;Independent$$

$$Q\left(F_{1}\cap F_{2}\right)>Q\left(F_{1}\right)+Q\left(F_{2}\right)\;Nonlinear\;enhancement$$

## Results and discussion

### Scenic area attractiveness

#### Scenic area popularity.

Combining spatial and temporal dimensions and social dimensions, the popularity of scenic spots is analyzed. On the one hand, mobile signaling data starts from the spatial and temporal dimension, focusing on the spatial distribution and time changes of tourists in the scenic spot. On the other hand, microblog check-in data starts from the social dimension, paying attention to the social interaction, interest preference and origin distribution of tourists. Through the comprehensive analysis of these two kinds of data, the spatial and temporal distribution characteristics, tourist flow rules and social behavior models of scenic area popularity were revealed, which provided a multi-dimensional research framework for the study of Dali scenic spot popularity.

**Fig 3:**
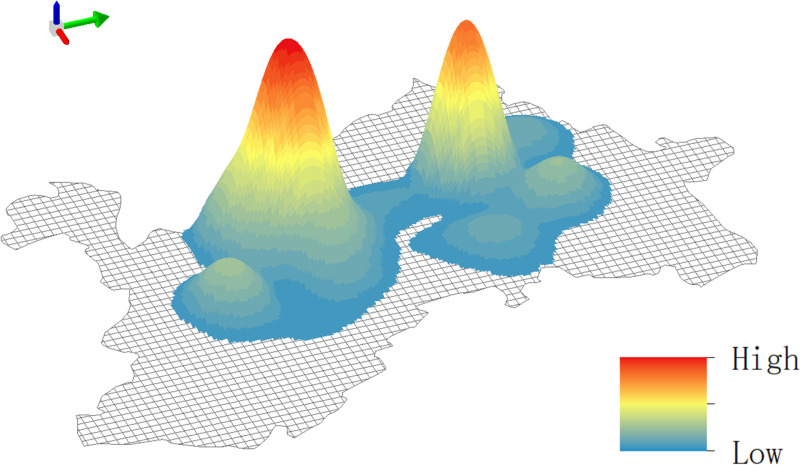
Kernel density distribution of scenic areas.

**(1) Analysis of mobile signaling data:** Fig 3 shows the results of a POI-based kernel density analysis. The overall distribution of scenic areas in Dali City showed a two-center and multi-point pattern, in which two scenic areas (i.e., Dali Ancient City and Xizhou Ancient Town) served as the core and numerous scenic areas showed a scattered pattern. Scenic areas with the low kernel density included Three Pagodas of Chongsheng Temple, Dali Ancient City, Cangshan Scenic Area, Tianlongbabu Studio City, Zhangjia Garden, and Southland City. Based on the Pearson correlation coefficients, we detected a significant correlation between the number of internal POIs and number of tourists in scenic areas (P = 0.739973), indicating a strong positive correlation between the number of internal POIs and the number of tourists in scenic areas. Therefore, for POI high-density scenic areas, scenic spots or cultural displays with local characteristics can be added, including setting up more interactive cultural experience projects or increasing special shopping, dining and other facilities, to further increase the stay time and consumption of tourists. For POI low-density scenic areas, strengthen resource development and publicity, better guide the flow of tourists, and promote the rational utilization and balanced development of tourism resources in Dali City.

**Fig 4:**
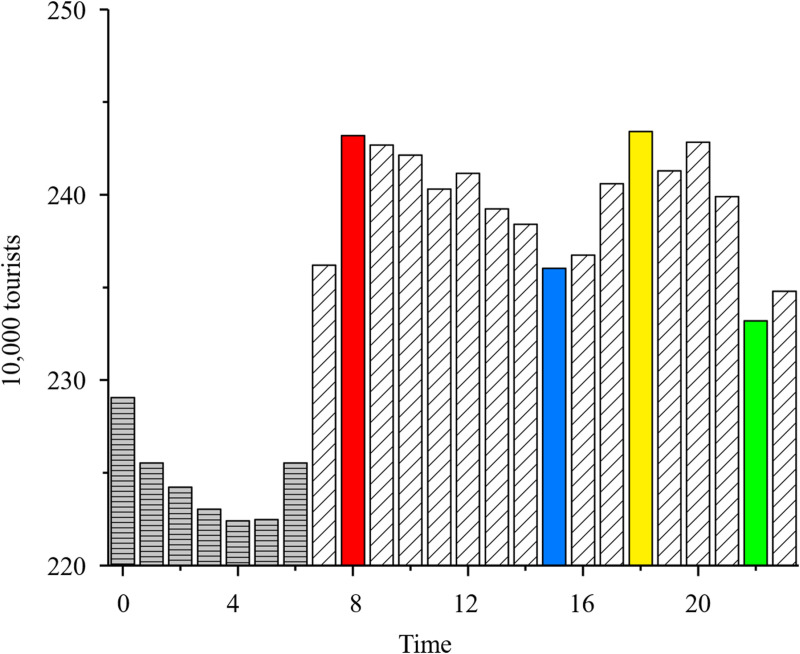
Change in the number of tourists per unit time.

Based on mobile signaling data, we analyzed changes in the daily number of tourists in different scenic areas and time periods. According to daily work and rest time, a time interval of 4 h was used; the first 8 h of each day were considered rest time, and four time periods (i.e., 8:00 to 12:00 (forenoon), 12:00 to 16:00 (afternoon), 16:00 to 20:00 (evening), and 20:00 to 24:00 (night)) were used as active periods. As shown in Fig 4, the most obvious changes in the number of tourists per unit time occurred at 8:00, 15:00, 18:00, and 22:00 during the four time periods. Around 8:00 and 18:00 were peak periods, during which it is recommended to take appropriate flow control measures, including increasing entry guidance and improving reception capacity. At around 15:00 and 22:00 were trough, it is recommended to carry out cleaning and maintenance of the scenic area to improve operational efficiency and tourist experience.

**Fig 5:**
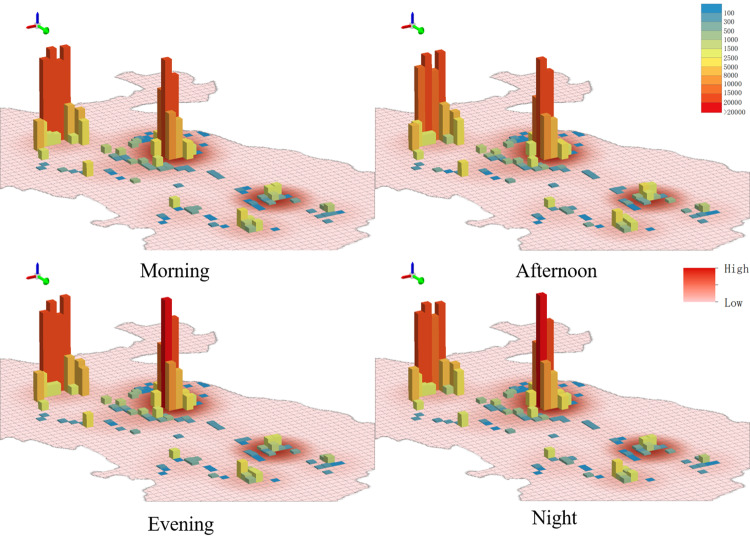
Scenic area popularity in different time periods.

Fig 5 shows the spatiotemporal distribution of tourists during four time periods. Dali Ancient City and southern Erhai Scenic Area had the highest degree of tourist aggregation. Moreover, the later the time, the higher the degree of tourist aggregation in Dali Ancient City, showing a striking contrast with the number of tourists in nearby scenic areas. It further explains its important position in night tourism and leisure life, the scenic area can optimize night tourism services, increase night cultural performances or activities, and further attract tourists to participate. The number of tourists in southern Erhai Scenic Area was large but showed little variation, indicating a relatively stable popularity across time periods, and it is suitable to develop more all-weather tourism activities or services to enhance the all-day attraction of the scenic area.

**Fig 6:**
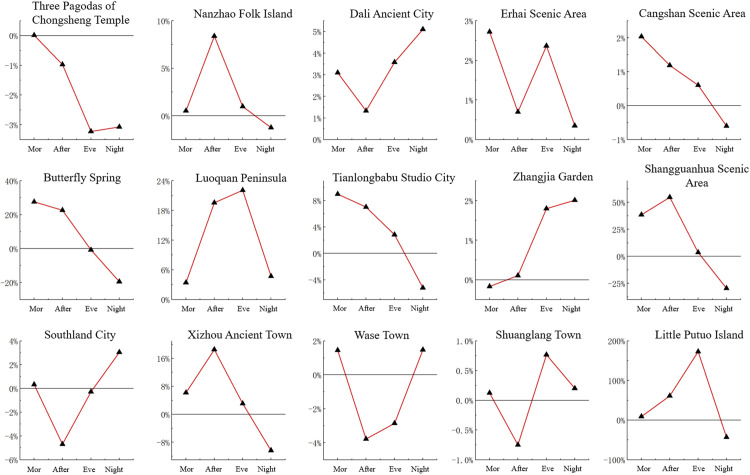
Changes in the number of tourists in different scenic areas and time periods.

Using the average number of tourists per scenic area within 24 h as a benchmark, we evaluated the changes in the number of tourists in different scenic areas across four time periods (Fig 6). The results summarized in Fig 6 and scenic area popularity in Fig 5 reveal several key findings. First, Dali Ancient City and Erhai Scenic Area attracted the largest number of tourists. In particular, the number of tourists increased from afternoon to night and reached a peak at night in Dali Ancient City. It is recommended to increase night security measures, and increase the services of catering, entertainment, public transportation and other facilities to ensure a comfortable experience for tourists. A similar trend in the number of tourists was observed in the Erhai Scenic Area, as shown in Fig 4, with the largest values in the morning and smallest values at night. The scenic area can consider introducing night tours, such as night boats or night light displays, to enhance its night appeal and further balance the flow of tourists in one day. Second, after these two scenic areas, Three Pagodas of Chongsheng Temple, Southland City, and Zhangjia Garden had the largest number of tourists. In Three Pagodas of Chongsheng, the number of tourists reached a peak in the morning, followed by a decrease owing to the limited opening time. In Southland City and Zhangjia Garden, the number of tourists also showed a significant upward trend, suggesting that the distance between scenic areas influences scenic area popularity, namely (i.e., a popular scenic area can drive the development of neighboring scenic areas). Third, Cangshan Scenic Area and Tianlongbabu Studio City, which are adjacent, showed similar trends in the number of tourists, with peak visitation occurring in the morning, followed by a gradual decline throughout the day. In view of this feature, the two scenic areas can consider withdrawing from characteristic activities in the afternoon or evening, such as walking, local culture display, the development of new routes or short-term characteristic activities, etc., to enhance the stay time and visit willingness of tourists and avoid a single concentration of tourists. Fourth, Xizhou Ancient Town and Shuanglang Town shared similar number of tourists. The number of tourists in Xizhou Ancient Town reached a peak in the afternoon, and the number of tourists in Shuanglang Town reached a peak in the evening. These scenic areas can further attract more visitors by extending or advancing the peak flow period, extending the afternoon period to evening, and advancing the evening period to afternoon, including extending the opening hours of catering and entertainment facilities, and launching exclusive activities in the evening period (sunset viewing, music and art exhibitions, etc.). Fifth, in Wase Town, the number of tourists in the morning was similar to that in the evening; however, there was an outflow of tourists in the daytime, indicating that there are a large number of residents near the scenic area. In other scenic areas, the basic quantity of tourists was relatively small; however, activity levels were typically higher in the daytime than at night.

Through the analysis of mobile signaling data, not only reveals the heat distribution characteristics of major tourist attractions in Dali City, but also provides data support for tourist flow management and resource optimization. Judging from the high popularity of Dali Ancient City and Erhai Scenic Area, these two scenic areas have natural advantages in tourist attraction, therefore, emphasis should be placed on improving the infrastructure and service level of these two scenic areas to cope with the growing number of tourists. At the same time, other scenic areas are less popular, it is recommended to strengthen the promotion and resource allocation of these scenic areas through reasonable tourist guidance and diversion measures, especially the Cangshan Scenic Area and Tianlongbabu Studio City and other scenic areas, can realize the balanced development of regional tourism through the radiation effect of Dali Ancient City and Erhai Scenic Area. Combined with the analysis of temporal and spatial data, scenic area managers can formulate more targeted management plans according to the tourist flow of different periods, improve tourist experience, and rationally allocate scenic area resources. The analysis provides systematic support for the regional tourism coordination of Dali scenic area, which is conducive to the long-term development and sustainable growth of Dali tourism industry.

**Fig 7:**
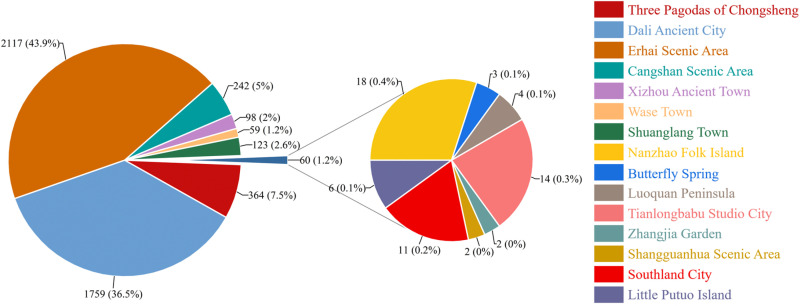
Microblog check-in number of tourists in different scenic areas.

**(2) Analysis of microblog check-in data:** Fig 7 shows the number of tourists based on microblog check-in data for different scenic areas of Dali City. The number of microblog check-ins was highest in Erhai Scenic Area and Dali Ancient City (collectively accounting for 80% of check-ins) and was substantially lower in eight scenic areas (including Nanzhao Folk Island, Butterfly Spring, Luoquan Peninsula, Tianlongbabu Studio City, Zhangjia Garden, Shangguanhua Scenic Area, Southland City, and Little Putuo Island) (collectively accounting for only 1.2%), with the lowest numbers in the Shangguanhua Scenic Area and Zhangjia Garden. In terms of the quantity of tourists based on microblog check-in data, Dali Ancient City and Erhai Scenic Area showed greater popularity than those of other scenic areas. In order to cope with the growing number of tourists in popular scenic areas, it is suggested to strengthen the management and facility construction of scenic areas, such as guidance systems, service points and toilets, to avoid the inconvenience caused by excessive concentration of tourists. At the same time, consider ways to guide the flow of tourists and encourage tourists to visit other scenic areas, including the launch of joint ticketing, special activities or regional tourism packages, so as to achieve a balanced distribution of tourism resources.

**Fig 8:**
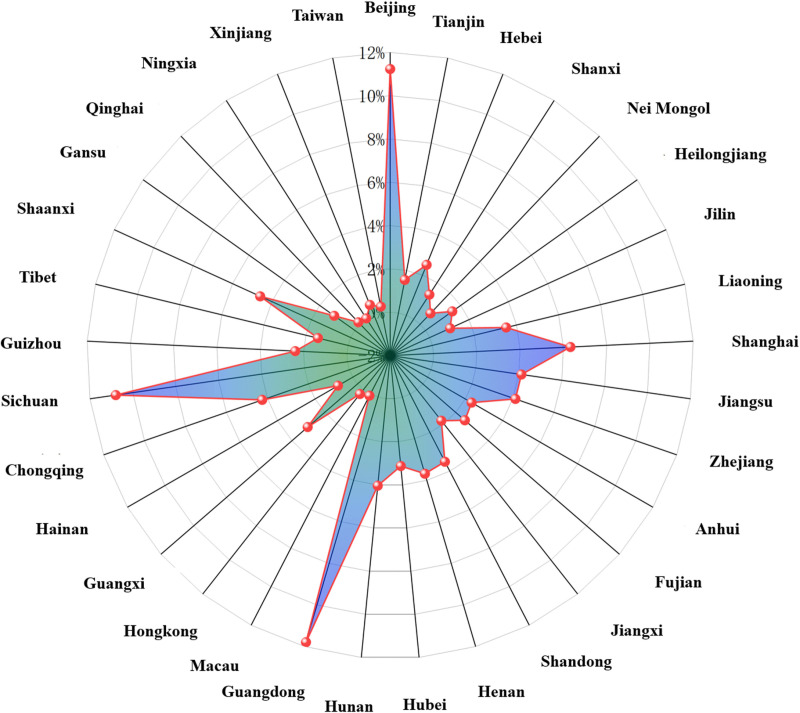
Quantity of tourists based on microblog check-in data for non-Yunnan provincial regions.

A statistical analysis of tourist sources showed that nearly half of total tourists come from Yunnan Province, with tourists in Dali City accounting for 37.46%. In terms of marketing strategy, we can consider increasing the publicity of these places, especially Kunming and Dali City in Yunnan Province, to attract more tourists through the word-of-mouth effect of local tourists, including carrying out local preferential activities and joint publicity, so as to enhance the tourism participation of local residents. Among the non-Yunnan provincial regions (Fig 8), Beijing, Sichuan, and Guangdong contributed the largest quantity of tourists (each accounting for >10%), followed by Shanghai, Jiangsu, Zhejiang, Hunan, Chongqing, and Shaanxi (each accounting for approximately 5%). At the same time, tourists from other provinces can vigorously develop promotion channels combining online and offline, and launch customized tourism projects to further enhance the attractiveness of tourists from outside the province.

The distribution of tourist sources based on check-in data for different scenic areas of Dali City. Yunnan Province contributed a large quantity of tourists for scenic areas of Dali City; in particular, more tourists were from Dali City and Kunming City than from non-Yunnan provincial regions. The tourist sources for Dali Ancient City and Erhai Scenic Area were widely distributed, covering nearly all parts of China (e.g., Sichuan, Guangzhou, and Beijing). For these scenic areas, precision marketing can be carried out through local tourism exhibitions, travel agency cooperation, social media and other channels to launch customized tourism services that meet the needs of tourists from all over the world. The tourist sources for Butterfly Spring, Luoquan Peninsula, Shangguanhua Scenic Area, and Little Putuo Island were not highly diverse (mainly including Yunnan Province and Beijing City). The tourist sources for Zhangjia Garden were limited to Dali City. For these scenic areas with relatively single tourists, we can consider attracting more foreign tourists to visit by joint marketing, enhancing the visibility of scenic areas, holding special activities, etc. By promoting more short-distance tours and weekend tours, etc., taking advantage of the convenience of tourists in the province, we can further enhance the frequency of visiting scenic areas.

The microblog check-in data further provided a clear picture of the tourist origin distribution in Dali scenic area. According to the analysis results, Dali Ancient City and Erhai Scenic Area are undoubtedly the most concentrated and popular scenic areas for tourists. These two scenic areas attract tourists from all over the country, especially tourists from the economically developed areas in and around the province, showing their wide appeal. In view of this phenomenon, the future should strengthen the tourist flow management of these two scenic areas, enhance the reception capacity. For tourists in and around the province, more tourism products that meet the needs of local residents can be designed to enhance the potential of the local market. For tourists from other provinces, especially those from economically developed areas such as Beijing and Guangdong, they can enhance their travel experience through precision marketing and further enhance the visit rate of tourists from other provinces. On the whole, the analysis of microblog check-in data provides an important basis for the target market positioning, tourist flow management and resource optimization of Dali scenic area, which is conducive to further improving the sustainable development of Dali’s tourism industry.

Table 3 describes the results for scenic area popularity calculated using the gravity model. The 15 scenic areas in descending order of popularity were as follows: Erhai Scenic Area, Dali Ancient City, Three Pagodas of Chongsheng Temple, Cangshan Scenic Area, Shuanglang Town, Xizhou Ancient Town, Shangguanhua Scenic Area, Wase Town, Nanzhao Folk Island, Little Putuo Island, Luoquan Peninsula, Butterfly Spring, Tianlongbabu Studio City, Zhangjia Garden, and Southland City. The popularity of Erhai Scenic Area and Dali Ancient City is far ahead, which shows the important position of these two scenic areas in the hearts of tourists. The Three Pagodas of Chongsheng and Cangshan Scenic Area are in the high heat zone, while Southland City, Zhangjia Garden and Butterfly Spring, although there is a certain number of tourists, but the heat is low, may need to further optimize the allocation of tourism resources to enhance the popularity of scenic areas.

**Table 3:** Scenic area popularity.

Scenic area	Popularity	Scenic area	Popularity	Scenic area	Popularity
Three Pagodas of Chongsheng Temple	11.638	Butterfly Spring	1.163	Southland City	0.918
Nanzhao Folk Island	1.856	Luoquan Peninsula	1.249	Xizhou Ancient Town	4.439
Dali Ancient City	43.199	Tianlongbabu Studio City	1.104	Wase Town	2.686
Erhai Scenic Area	48.817	Zhangjia Garden	0.992	Shuanglang Town	4.607
Cangshan Scenic Area	7.110	Shangguanhua Scenic Area	4.387	Little Putuo Island	1.745

#### Scenic area satisfaction.

**Fig 9:**
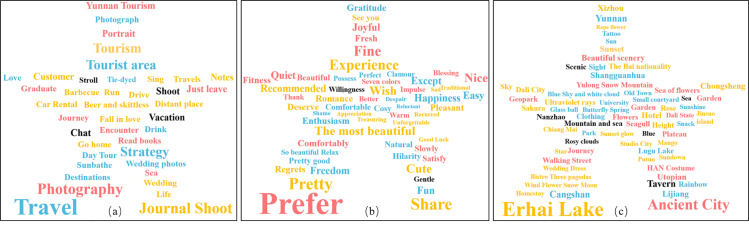
Word cloud map showing high-frequency words related to (a) the destination, (b) adjectives, and (c) the check-in location.

In this study, microblog check-in text data were analyzed using ROST-CM6. The top 1,000 most frequent words related to “purpose,” “adjective,” and “check-in place” were identified and used to generate a word cloud (Fig 9). The words with high frequency in Fig 9a include “travel”, “travel photography”, “tourism”, etc., indicating that the purpose of most tourists is to travel or shoot for leisure. At the same time, it also proves the reliability of microblog check-in data, indicating that Dali is attractive as a tourist destination, attracting a large number of tourists who have needs for travel and shooting. In Fig 9b, words with high frequency include “like”, “most beautiful”, “good-looking”, etc. Most of them are positive words, indicating that tourists have a high overall satisfaction with the scenic spot and the scenic area has a good reputation. In Fig 9c, words with high frequency include “Erhai”, “Ancient city” and “Chongsheng Temple”, etc. These high-frequency words show the words frequently mentioned by tourists in the check-in texts, and further confirm the positive correlation between the popularity and popularity of scenic spots.

Table 4 describes the results of a quantitative sentiment analysis for the 15 scenic areas. In terms of scenic area satisfaction, the 15 scenic areas were ranked in descending order as follows: Butterfly Spring, Tianlongbabu Studio City, Zhangjia Garden, Shangguanhua Scenic Area, Southland City, Shuanglang Town, Wase Town, Xizhou Ancient Town, Dali Ancient City, Three Pagodas of Chongsheng Temple, Erhai Scenic Area, Cangshan Scenic Area, Nanzhao Folk Island, Little Putuo Island, and Luoquan Peninsula. Overall, the satisfaction of each scenic spot is generally high. However, it should be noted that for some scenic areas with full satisfaction scores, there may be some bias due to the small sample data and the polarization effect of social media. In order to draw more representative and reliable conclusions, the future will consider expanding the sample size, integrating feedback data from more dimensions, and further screening and correction of social media data.

**Table 4:** Scenic area satisfaction.

Scenic area	Satisfaction	Scenic area	Satisfaction	Scenic area	Satisfaction
Three Pagodas of Chongsheng Temple	96.44	Butterfly Spring	100	Southland City	100
Nanzhao Folk Island	94.74	Luoquan Peninsula	75	Xizhou Ancient Town	97.98
Dali Ancient City	96.53	Tianlongbabu Studio City	100	Wase Town	98.31
Erhai Scenic Area	96.27	Zhangjia Garden	100	Shuanglang Town	98.37
Cangshan Scenic Area	95.06	Shangguanhua Scenic Area	100	Little Putuo Island	83.33

#### Scenic area attractiveness.

Scenic area popularity and satisfaction were combined (with equal weights) to calculate scenic area attractiveness (Table 5). In terms of scenic area attractiveness, the 15 scenic areas were ranked in descending order as follows: Erhai Scenic Area, Dali Ancient City, Three Pagodas of Chongsheng Temple, Cangshan Scenic Area, Shuanglang Town, Shangguanhua Scenic Area, Xizhou Ancient Town, Wase Town, Nanzhao Folk Island, Butterfly Spring, Tianlongbabu Studio City, Zhangjia Garden, Southland City, Little Putuo Island, and Luoquan Peninsula.

**Table 5:** Scenic area attractiveness.

Scenic area	Attractiveness	Scenic area	Attractiveness	Scenic area	Attractiveness
Three Pagodas of Chongsheng Temple	10.641	Butterfly Spring	5.5815	Southland City	5.459
Nanzhao Folk Island	5.665	Luoquan Peninsula	4.3745	Xizhou Ancient Town	7.1185
Dali Ancient City	26.426	Tianlongbabu Studio City	5.552	Wase Town	6.2585
Erhai Scenic Area	29.222	Zhangjia Garden	5.496	Shuanglang Town	7.222
Cangshan Scenic Area	8.308	Shangguanhua Scenic Area	7.1935	Little Putuo Island	5.039

From the results of scenic attraction, it can be found that the attractiveness of Erhai Scenic Area and Dali Ancient City is much higher than other scenic spots, which reflects that these two scenic areas not only dominate in the number of tourists, but also highly recognized by tourists for their experience. Secondly, although the popularity of some scenic areas is low, but its high satisfaction also improves the attractiveness score, including Xizhou Ancient Town, Shuanglang Town, Wase Town and Shangguanhua Scenic Area; On the contrary, Cangshan Scenic Area and the Three Pagodas of Chongsheng have higher popularity but lower satisfaction. Finally, for the low-attraction scenic areas, mainly because the popularity and satisfaction of scenic areas are not outstanding, including Luoquan Peninsula, Nanzhao Folk Island and Little Putuo Island. Among them, Butterfly Spring and Southland City are full marks in satisfaction, but due to their lower heat, resulting in lower overall attractiveness.

The popularity and satisfaction of the scenic area are the two key factors that determine the attraction of the scenic area. The popularity of the scenic area reflects the popularity of the scenic area and the attention of tourists, while the satisfaction reflects the experience of tourists in the scenic area. On the one hand, for scenic areas with low popularity but high satisfaction, we can consider increasing their popularity through publicity and improving tourists’ sense of experience, so as to further enhance their attractiveness. On the other hand, for scenic spots with high popularity but low satisfaction, emphasis should be placed on improving the quality of tourist service and the configuration of infrastructure to enhance the overall experience of tourists. All in all, to enhance the attraction of scenic spots, it is not only necessary to increase the popularity of scenic areas, but also to ensure the high satisfaction of tourists, forming a virtuous circle to attract more tourists and increase the market competitiveness of scenic areas.

### Influencing factors and correlations

We selected 13 candidate factors related to scenic area attractiveness covering different aspects (e.g., food, accommodation, travel, and shopping). Subjective factors included scenic area opening time, ticket prices, degree of praise, travel distance, and play time. Objective factors included the scenic area grade, scenic area type, number of internal POIs, and grid area. Further, the number of ambient POIs included the numbers of catering-related, accommodation-related, shopping-related, and traffic-related POIs. Data for the degree of praise and play time were provided by the Mafengwo APP. Today, high-speed rail is the preferred mode of travel and is highly convenient. Therefore, the travel distance refers to the distance from a high-speed rail station to a scenic area; there were two types of scenic areas, namely, human and natural landscapes.

**Fig 10:**
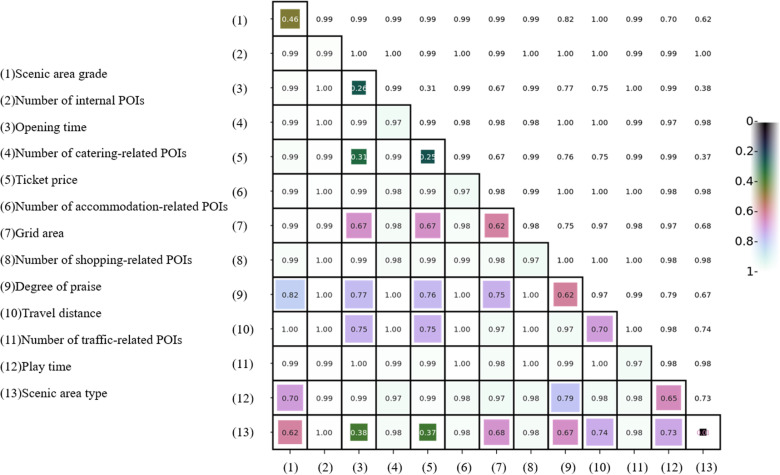
Q values for the interaction effects of influencing factors on scenic area attractiveness [51].

The influencing factors were discretized, and the discretization scheme with the largest Q value was selected (Fig 10). The higher the decision-making power of a factor, the more significant its effect on scenic area attractiveness. In terms of Q values, the 13 candidate factors were ranked in descending order as follows: number of internal POIs (0.993), number of traffic-related POIs (0.973), number of accommodation-related POIs (0.971), number of shopping-related POIs (0.969), number of catering-related POIs (0.968), travel distance (0.703), play time (0.653), grid area (0.620), degree of praise (0.615), scenic area grade (0.458), scenic area opening time (0.259), ticket price (0.250), and scenic area type (0.007).

The decision-making power differed significantly among the influencing factors for scenic area attractiveness in Dali City. The number of internal POIs had the most significant effect on scenic area attractiveness; this finding showing that the more POIs in a scenic area, the more tourists and the higher the attractiveness. Therefore, it is suggested to pay attention to the development of natural landscape in the construction of Dali scenic area, avoid the homogeneity of artificial classics, in order to enrich the types and contents of scenic areas, and further enhance the diversity and attraction of scenic areas. The number of ambient POIs (including traffic-related, catering-related, accommodation-related, and shopping-related POIs) had significant effects on scenic area attractiveness, and the top five influencing factors were all statistically significant. First of all, considering that the number of tourists in Dali City is increasing year by year, and the carrying capacity of the original transportation facilities is challenged, it is suggested to increase the optimization and construction of the transportation network around the scenic area, especially around the popular scenic areas, to set up more public transportation lines, tourist dedicated buses and shared bicycles, so as to shorten the travel time and cost of tourists. At the same time, Dali City with unique natural conditions and rich folk culture, characteristic homestays have become an important attraction factor. To this end, it is suggested to vigorously develop high-quality services such as characteristic homestays and boutique hotels, and strengthen the publicity of local special cuisines, improve the quality of catering services, and further strengthen the cultural experience of tourists. Among the subjective factors, travel distance and play time influenced scenic area attractiveness significantly; in particular, a shorter travel distance and longer play time led to higher scenic area attractiveness. In this regard, it is suggested to strengthen the rapid connection between scenic areas and transportation hubs (such as high-speed rail stations, airports), and design efficient direct transportation systems. In addition, efficient half-day or day-trip routes suitable for short-distance tourists should be developed to meet the needs of different tourists. At the same time, by increasing diversified play activities, such as Erhai cycling, Cangshan hiking, tie-dye production and other cultural experience projects, to enhance the stay time of tourists in the scenic area and the overall experience. It is suggested to strengthen the word-of-mouth construction and information feedback mechanism of tourists, encourage tourists to share their play experience through social media, tourism platforms and other channels, and promptly adopt suggestions from tourists. It is helpful to improve the online evaluation of scenic areas and promote the positive publicity and word-of-mouth effect of scenic areas. Among the objective factors, grid area and scenic area grade affected scenic area attractiveness significantly; in particular, a larger grid area and higher scenic area grade led to higher scenic area attractiveness. Therefore, it is recommended to pay attention to expanding open space and increasing public facilities such as walking paths in the development process of scenic areas to enhance tourists’ sense of closeness and play experience. In addition, the level of scenic areas also has a great impact on attractiveness, especially scenic areas with rich cultural and historical resources, should strengthen hardware facilities, tourist services and environmental protection, so as to improve the level of scenic areas and the overall attractiveness. Finally, the type of scenic area has the least influence on the attraction of scenic areat. As Dali City has a low-latitude plateau monsoon climate, the temperature difference between the four seasons is small, and the natural landscape has a unique diversity, so the type of scenic area has no significant influence on the attraction of the scenic area. In order to make full use of Dali’s climatic and geographical advantages, it is suggested to strengthen the combination of natural landscape and cultural heritage, and promote the integration of ecological tourism and cultural tourism. Specifically, diversified tourism products can be introduced according to the four seasons, such as outdoor hiking in spring and summer, hot springs and skiing in autumn and winter, to attract year-round tourists.

The interactive decision-making power of the influencing factors for scenic area attractiveness was greater than the decision-making power of individual factors. It shows that the attraction of scenic spots is not only determined by a single factor, but also by the interaction of many factors. $Q_{scenic\;area\;grade}\cap Q_{opening\;time},\;Q_{Scenic\;area\;grade}\cap Q_{ticket\;price},\;Q_{scenic\;area\;grade}\cap Q_{scenic\;area\;type},\;Q_{opening\;time}\cap Q_{play\;time},\;Q_{ticket\;price}\cap Q_{play\;time},\;Q_{ticket\;price}\cap Q_{scenic\;area\;type}$ exhibited nonlinear enhancement, the interaction of different factors brings about a nonlinear enhancement effect that exceeds the sum of their individual effects, because their effects are dynamic and variable, and need to be optimized by more complex strategies. For example, the interaction between the level of scenic areas and the opening hours means that the opening hours of high-grade scenic areas are extended, and the stay time and satisfaction of tourists may be significantly increased, but the effect of extending the opening hours of low-grade scenic areas is not obvious, and even increases the management cost. Therefore, for high-grade scenic areas, the extension of opening hours should be considered during holidays or in peak tourist seasons, and for low-grade scenic areas, the extension of opening hours may need to be combined with other factors (such as activity planning) to enhance the effect. The interaction with ticket prices may mean that high-class scenic areas have some flexibility in pricing and can better attract tourists by setting the right ticket price, while low-class scenic areas should avoid excessive ticket pricing and ensure that the price is reasonable and competitive. The importance of interacting with the types of scenic areas is that different types of scenic areas have great differences in tourist expectations and needs. Scenic areas should set up services and activities that meet the needs of tourists according to their own type characteristics, such as cultural scenic areas can increase cultural experience activities, and natural scenic areas can increase ecological tourism projects to maximize the synergy between grades and types. The interaction between ticket price and travel time means that high-ticket scenic areas will make tourists pay more attention to travel time and quality, reflecting the behavioral difference between price-sensitive tourists and high-spending tourists. In the interaction between play time and opening time, the longer the opening time of the scenic area, the play time may increase accordingly. The scenic area manager can extend the opening time during off-peak hours to improve the play experience of tourists. During peak hours, adjust the play items appropriately to avoid tourists feeling tired due to too long play time. In the interaction with the ticket price, high ticket prices often need to provide longer play time and more diversified activities to meet the expectations of tourists, therefore, scenic areas can extend the play time (such as increasing night tour items, etc.) to match the high-ticket prices and enhance the value perception of tourists. For the interaction between ticket prices and types of scenic areas, scenic areas should formulate ticket strategies according to their own types. Cultural scenic areas can enhance ticket acceptance through rich cultural experiences and unique educational and cultural values, while natural scenic areas should avoid excessive ticket pricing to ensure that the burden of tourists is not too heavy. And other interactions showed double-factor enhancement. The rest are two-factor enhancement types, that is, the combination of two factors will bring the improvement of decision-making power, suggesting that managers can maximize the effect of some factors by combining optimization strategies. For example, the interaction between the level of scenic area and the number of POI (such as transportation, catering, accommodation, shopping) shows that high-grade scenic areas are usually accompanied by more POI of surrounding facilities, and managers should pay attention to the construction of surrounding facilities to improve the quality of catering and accommodation around and the convenience of transportation. The interaction between favorable rating and travel time indicates that tourists in scenic areas with high satisfaction are usually willing to stay longer. Therefore, paying attention to tourist experience and optimizing service facilities can form a positive cycle for the attraction of scenic spots.

Based on the analysis results of factor interaction, scenic area managers can arrange scenic area resources more scientifically and formulate reasonable opening hours, ticket pricing and scenic area facility configuration according to different tourist needs, which provides an important reference for the formulation of future scenic area management policies. Considering that the attraction of scenic areas is affected by a variety of dynamic factors, future research plans to further introduce more advanced models and diversified data to explore the interaction of multi-dimensional factors such as seasonal changes of scenic areas, the number of tourists during holidays, and the behavioral characteristics of different types of tourists, so as to provide more accurate and flexible decision-making basis for scenic area managers.

## Conclusions

Based on POI, mobile signaling, and microblog check-in data, we analyzed the popularity of scenic areas in Dali City using various methods (e.g., a kernel density analysis, hotspot analysis, and gravity model). We also performed a sentiment analysis using microblog text data and determined scenic area satisfaction using ROST-CM6. Finally, we determined scenic area attractiveness based on estimates of scenic area popularity and satisfaction. Using the GeoDetector method, we examined the effects of subjective and objective factors and the number of ambient POIs on the scenic area attractiveness of Dali City. Our findings are summarized as follows.

Distribution and heat characteristics of scenic areas. Dali City’s scenic areas show a distribution pattern of “two-center and multi-point” scenic areas centered on Dali Ancient City and Xizhou Ancient Town. Dali Ancient City and Erhai Scenic area are the core areas where tourists gather. Among them, the night heat of Dali Ancient city is higher, which reflects its diversified leisure consumption needs to meet the advantages of one-stop service. More broadly, night tourism is increasing revenue in scenic areas, enhancing urban vitality and openness, and promoting urban economic growth.

Second, sources of tourists and characteristics of scenic areas. The tourists of Dali City mostly come from Yunnan Province and its neighboring provinces as well as cities in economically developed coastal regions. The tourist sources for Dali Ancient City and Erhai Scenic Area were widely distributed throughout China. The tourist sources for Butterfly Spring, Luoquan Peninsula, Shangguanhua Scenic Area, and Little Putuo Island were relatively limited. The geographical location of a scenic area, its popularity and the preference of tourists have an important impact on the attraction of a scenic area.

Third, micro-blog analysis and tourist satisfaction. The emotion analysis of micro-blog shows that tourists’ emotions towards Dali scenic areas are mostly positive, and the high-frequency words are related to the travel destination and the time-card place, which reflects that tourists’ satisfaction and popularity of scenic area are closely related.

Fourth, determinants of attraction of scenic areas. Research shows that the attraction of scenic areas is mainly affected by the number of scenic spots and the number of surrounding facilities (POI). The more scenic spots in the scenic area, the stronger the attraction, and the perfection of the surrounding facilities also has a significant impact on the attraction, while the type of scenic area has a small impact on the attraction. In addition, the interaction of multiple factors significantly improves the decision-making power, indicating that the difference of Dali scenic attraction is the result of the joint action of multiple factors.

Through the quantitative analysis of the popularity of scenic areas, tourist satisfaction and attraction, the research reveals the main influencing factors of scenic attraction and their interaction modes, which provides the following practical significance for urban tourism development and scenic area management: First of all, in the optimization of scenic resource allocation. The number of scenic spots in the scenic area and the abundance of surrounding facilities have a significant impact on the attraction of the scenic area. Therefore, scenic area managers should pay attention to the diversification of scenic spots and the improvement of surrounding facilities (such as catering, accommodation, transportation), especially in areas with large tourist volume such as Dali Ancient City and Erhai Scenic Spot, should further improve service facilities and enhance tourist experience. Second, promote the development of night economy. The high night heat in Dali Ancient City indicates that its night economy has a positive impact on the attraction of scenic areas. Policy makers should encourage scenic areas and surrounding commercial areas to develop a variety of night activities and cultural experiences to attract tourists to stay longer, thereby enhancing the economic efficiency of scenic areas and urban vitality. Then, precise marketing. Different scenic areas attract different sources and needs of tourists, so scenic areas should formulate accurate marketing strategies according to their own characteristics and tourist source distribution. For example, scenic areas targeting tourists from within the province can focus on promoting cultural features and leisure projects, while scenic areas targeting tourists from across the country should strengthen their visibility and brand building. Finally, improve tourist satisfaction. Emotion analysis through social platforms such as micro-blog can help scenic area managers better understand the needs and sexual tendencies of tourists, so as to improve the service quality and enhance the overall satisfaction and return rate of tourists.

## Discussion

This paper analyzes the attractiveness of scenic areas quantitatively by combining mobile phone signaling, microblog check-in and other temporal and spatial data, which is conducive to providing comparison and reference for the research on scenic area attractiveness under the integration of multi-source data. In recent years, the research on scenic attraction has gradually turned to multi-dimensional and interactive analysis [19–21]. Many scholars have realized that the attraction of scenic area is the effect of multiple factors. For example, the attraction of scenic area not only depends on its natural and historical cultural resources, but also is comprehensively affected by tourists’ experience, service quality, scenic area facilities and other aspects. Through the quantitative analysis of multi-source temporal and spatial data, this study further verified this view and revealed the impact of interaction between different factors on scenic attraction. In addition, compared with the traditional single factor analysis model, the interaction analysis of multiple factors reveals the enhancement effect between different factors, which provides more accurate decision-making basis for scenic area managers.

There are still some limitations in this study, which may affect the universality and accuracy of the research conclusions to some extent, including the following aspects: First, the time range of data collection is limited. The mobile signaling data only includes one working day, ignoring the research on the space-time movement of holiday crowds. During the peak period, the travel patterns and behavioral characteristics of tourists may be very different from those on weekdays. Therefore, future studies should expand the scope of data collection to cover different time periods (such as holidays, weekends, peak periods, etc.) to more comprehensively understand the dynamic changes in tourist behavior and attraction of scenic areas. Second, sentiment analysis has certain limitations. In the satisfaction analysis of scenic areas, the user groups and emotional expression of micro-blog platform are one-sided and subjective. Therefore, in future studies, it is further considered to combine more data such as service reviews, picture labels, travel notes and images, and follow the research model of “multi-source data, mixed methods, cross-research and team collaboration” [28] to improve the accuracy and comprehensiveness of sentiment analysis through multi-modal analysis. Third, the lack of qualitative analysis. This study mainly relies on quantitative analysis to study spatio-temporal data, future research studies will consider combining qualitative research methods, including in-depth interviews, focus groups and participant observation, to further explore tourists’ motivation, emotional expression and cognition of scenic areas, so as to more comprehensively understand the composition of scenic attraction. Finally, the complexity and explanatory power of the model. Although the GeoDetector model has some advantages in examining factor interactions, it lacks in-depth discussion of causal mechanisms. In future studies, more complex models will be introduced to deeply analyze the influence mechanism of various factors on scenic attraction from the perspective of causality.

This study mainly analyzes the scenic areas of Dali City. Considering the uniqueness of Dali City (plateau monsoon climate, rich natural and cultural resources), the applicability of these conclusions may be limited. Although Dali is similar to Lijiang, Shangri-La and other cities in terms of tourism resources and geographical characteristics, the scenic attraction of other small and medium-sized tourist cities may be different due to factors such as resource distribution, development stage and tourist demand. In addition, geographical and cultural differences may also affect the determinants of attraction, for example, the appeal of coastal cities may rely more on waterfront views and international amenities, while plateau or mountain cities focus on natural landscapes and leisure experiences. Therefore, the research conclusions need to be adjusted accordingly under different geographical backgrounds and cultural conditions to adapt to the actual situation of urban development.

## References

[1] Leiper N. Tourist attraction systems. Ann Tour Res. 1990;17(3):367–84.

[2] Yangzhou Hu, Ritchie JRB. Measuring destination attractiveness: a contextual approach. J Travel Res. 1993;32(2):25–34. doi: 10.1177/004728759303200204

[3] Mehmetoglu M, Abelsen B. Examining the visitor attraction product: a case study. Tour Anal. 2005;9(4):269–84.

[4] Crampon L. Gravitational model approach to travel market analysis. J Mark. 1966;30(2):27–31.

[5] Wolfe RI. The inertia model. J Leis Res. 1972;4(1):73–6.

[6] Chang JR, Chang B. The development of a tourism attraction model by using fuzzy theory. Math Probl Eng. 2015;2015:1–10.

[7] Barros C, Botti L, Peypoch N, Elisabeth R, Bernardin S, Assaf A. Performance of French destinations: tourism attraction perspectives. Tour Manag. 2011;32(1):141–6.

[8] Harandi A, Mirzayan KP. Explaining health tourism attraction model: using classic grounded theory strategy. Urban Tour. 2017;4(1):87–98.

[9] Müller DK. The attractiveness of second home areas in Sweden: a quantitative analysis. Current Issues in Tourism. 2006;9(4–5):335–50. doi: 10.2167/cit269.0

[10] Weidenfeld A, Williams AM, Butler RW. Knowledge transfer and innovation among attractions. Ann Tour Res. 2010;37(3):604–26.

[11] Medina-Muñoz DR, Medina-Muñoz RD. The attractiveness of wellness destinations: an importance–performance–satisfaction approach. Int J Tour Res. 2014;16(6):521–33.

[12] Jensen Ø, Li Y, Uysal M. Visitors’ satisfaction at managed tourist attractions in Northern Norway: Do on-site factors matter? Tourism Management. 2017;63:277–86. doi: 10.1016/j.tourman.2017.06.025

[13] Boivin M, Tanguay G. Analysis of the determinants of urban tourism attractiveness: The case of Québec City and Bordeaux. J Destin Mark Manag. 2019;11:67–79.

[14] Zhang LY. Review on the study of tourism gravity models and its future. Geogr Res. 1989;1:76–87.

[15] Bao JG. An application of gravity model in tourism forecasting. Acta Scientiarum Naturalium Universitatis Sunyatseni. 1992;4:133–6.

[16] Wan N, Zhang L. A forecasting model of tourism destination market scale based on the gravitation model. J Henan Univ. 2010;40(1):4549.

[17] Wang HH. Tourism attraction analysis and theoretical model. Sci Econ Soc. 2003;4:44–7.

[18] Li S, Wang Z, Zhong Z. Gravity model for tourism spatial interaction: basic form, parameter estimation, and applications. Acta Geogr Sin. 2012;67(04):526–44.

[19] Tian M, Wu G. The comprehensive evaluation of tourism and anlies of effective factors for Louguantai in Xi’an city. Chin Agric Sci Bull. 2011;27(23):291–4.

[20] Shi X, Shi Q, Cai Z. An analysis of the influencing factors of urban leisure tourism attraction -- a case study of Nanjing. Econ Res Guide. 2019;2:97–9.

[21] Zhang H. An analysis of influencing factors of tourist attraction in historical towns -- taking Dayan ancient town of Lijiang as an example. Tourism Overview. 2018;12:126–7.

[22] Xu J, Wang P. Study on distribution characteristic of tourism attractions in international cultural tourism demonstration region in South Anhui in China. PLoS One. 2022;17(6):e0269948. doi: 10.1371/journal.pone.0269948 35731799 PMC9216503

[23] Wang M, Liu S, Wang C. Spatial distribution and influencing factors of high-quality tourist attractions in Shandong Province, China. PLoS One. 2023;18(7):e0288472. doi: 10.1371/journal.pone.0288472 37450422 PMC10348535

[24] Wang L, Liu J. Measuring on attractiveness of the red tourism scenic area based on the theory of fuzzy-ahp. J Nanjing Normal Univ. 2011;34(3):124–8.

[25] Xia JH. Study on the construction and promotion strategy of red tourism destination attraction evaluation system. Yangzhou University; 2014.

[26] Yuxin F, Yunxia T, Xiaoyu L. The network characteristics of classic red tourist attractions in Shaanxi province, China. PLoS One. 2024;19(3):e0299286. doi: 10.1371/journal.pone.0299286 38551967 PMC10980247

[27] Song SD. Research on the attractiveness of comprehensive parks in six districts of Beijing. North China University of Technology; 2020.

[28] Wu X. Research on evaluation system and construction strategy of national cultural tourism attraction. Res Dev. 2014;2014(1):131–4.

[29] Liu H, Hasan M, Cui D, Yan J, Sun G. Evaluation of tourism competitiveness and mechanisms of spatial differentiation in Xinjiang, China. PLoS One. 2022;17(2):e0263229. doi: 10.1371/journal.pone.0263229 35130280 PMC8820621

[30] Shi S, Li M, Li Z, Xi J. Spatial Heterogeneity and Influencing Factors of High-Grade Tourist Attractions in the Tibetan Plateau. Int J Environ Res Public Health. 2023;20(5):4650. doi: 10.3390/ijerph20054650 36901659 PMC10001576

[31] Zhu YQ. Research on beautiful rural landscape based on AVC tourism attraction evaluation index system. Soochow University; 2017.

[32] Jin L, Wang Z, Chen X. Spatial distribution characteristics and influencing factors of traditional villages on the Tibetan Plateau in China. Int J Environ Res Public Health. 2022;19(20):13170. doi: 10.3390/ijerph192013170 36293749 PMC9603369

[33] Li JY, Liu Y, Xiao WJ, Deng N, Xiong TT, Zhang K. Tourism big data research and application frontier series of pen talk. Tourism Forum. 2022;15(01):1–24.

[34] Liang W, Ahmad Y, Mohidin H. Spatial pattern and influencing factors of tourism based on POI data in Chengdu, China. Environ Dev Sustain. 2024;26(4):10127–43. 10.1007/s10668-023-03138-8 PMC10040312 37362977

[35] Jiang Y, Huang W, Xiong X, Shu B, Yang J, Li M. Investigating spatial patterns and determinants of tourist attractions utilizing POI data: a case study of Hubei Province, China. Heliyon. 2024;10(11). 10.1016/j.heliyon.2024.e32370 PMC11219313 38961968

[36] Peng R, Gao W. Spatial distribution pattern and driving mechanism of tourist attractions in Gansu Province based on POI data. PLoS One. 2023;18(10):e0292165. doi: 10.1371/journal.pone.0292165 37797069 PMC10553315

[37] Luo Y, Zhang X, Qin Y, Yang Z, Liang Y. Tourism Attraction Selection with Sentiment Analysis of Online Reviews Based on Probabilistic Linguistic Term Sets and the IDOCRIW-COCOSO Model. Int J Fuzzy Syst. 2020;23(1):295–308. doi: 10.1007/s40815-020-00969-9

[38] Li J, Cao B. Study on Tourism Consumer Behavior and Countermeasures Based on Big Data. Comput Intell Neurosci. 2022;2022:6120511. doi: 10.1155/2022/6120511 35909820 PMC9325599

[39] Chen X, Zhang A, Huang Z. Characteristics of tourists flow in scenic spots based on Weibo check-in big data: A case study of Zhongshan scenic area in Nanjing city. Econ Geogr. 2018;38(09):206–14.

[40] Wang D, Zuo X. Research on the attraction of urban scenic spots based on Weibo check−in data. Urban Geotech Investig Surv. 2020;6:32–7.

[41] Chen X, Li X, Guan J. Resident satisfaction-based updating strategies of old communities: a case study of Harbin demonstration communities. Areal Res Dev. 2019;38(04):85–91.

[42] Alfred R, Chen Z, Eboy OV, Luxuan Z, Renjie L. Analyzing trends in the spatial-temporal visitation patterns of mainland Chinese tourists in Sabah, Malaysia based on Weibo social big data. Heliyon. 2023;9(5):e15526. doi: 10.1016/j.heliyon.2023.e15526 37144192 PMC10151365

[43] Raun J, Ahas R, Tiru M. Measuring tourism destinations using mobile tracking data. Tour Manag. 2016;57:202–12.

[44] Wan Y, Zhang Y, Xie W, Ding Y, Tao Y, Zhu X. Research on spatiotemporal activity characteristics of tourists in scenic spots of Beijing based on mobile phone data. Beijing Surveying and Mapping. 2022;36(04):386–93.

[45] Gu QS, Zhang HP, Chen W, Xie Y. Hierarchical network structures and regional differentiations of tourist source destinations of Nanjing based on cellular signaling data. Sci Geogr Sin. 2019;39(11):1739–48.

[46] Zelenka J, Azubuike T, Pásková M. Trust model for online reviews of tourism services and evaluation of destinations. Adm Sci. 2021;11(2):34.

[47] Yan J, Yue J, Zhang J, Qin P. Research on Spatio-Temporal Characteristics of Tourists’ Landscape Perception and Emotional Experience by Using Photo Data Mining. Int J Environ Res Public Health. 2023;20(5):3843. doi: 10.3390/ijerph20053843 36900853 PMC10001129

[48] Lu J. Personalized recommendation algorithm of smart tourism based on cross-media big data and neural network. Comput Intell Neurosci. 2022;2022:9566766. doi: 10.1155/2022/9566766 35795765 PMC9251073

[49] Liu F, Sun D, Zhang Y, Hong S, Wang M, Dong J, et al. Tourist landscape preferences in a historic block based on spatiotemporal big data – A case study of Fuzhou, China. Int J Environ Res Public Health. 2022;20(1):83. doi: 10.3390/ijerph20010083 36612401 PMC9819072

[50] Wang JF, Xu CD. Geodetector: principle and prospective. Acta Geogr Sin. 2017;72(1):116–34.

[51] Fisher CD. Padlet: an online tool for learner engagement and collaboration. AMLE. 2017;16(1):163–5. doi: 10.5465/amle.2017.0055

